# Role of mTORC2 in biphasic regulation of brown fat metabolism in response to mild and severe cold

**DOI:** 10.1016/j.jbc.2021.100632

**Published:** 2021-04-15

**Authors:** Prasanna K.R. Allu, Esther Paulo, Ambre M. Bertholet, Gavin Situ, Seung-Hwan Lee, Yixuan Wu, Catherine E. Gleason, Bidisha Saha, Ajay Chawla, Biao Wang, David Pearce

**Affiliations:** 1 Department of Medicine, Division of Nephrology, University of California at San Francisco, San Francisco, California, USA; 2 Cardiovascular Research Institute, University of California at San Francisco, San Francisco, California, USA; 3 Department of Physiology, University of California at San Francisco, San Francisco, California, USA

**Keywords:** mTOR, UCP1, cold, thermogenesis, lipogenesis, gene expression, brown adipose tissue, adipocytes, BAT, brown adipose tissue, DNL, *de novo* lipogenesis, iBAT, interscapular brown adipose tissue, MEF, murine embryo fibroblast, PEG, polyethylene glycol, PKA, protein kinase A, SNS, sympathetic nervous system, TBP, TATA-binding protein, UCP1, uncoupling protein-1

## Abstract

Nonshivering thermogenesis is essential for mammals to maintain body temperature. According to the canonical view, temperature is sensed by cutaneous thermoreceptors and nerve impulses transmitted to the hypothalamus, which generates sympathetic signals to ß-adrenergic receptors in brown adipocytes. The energy for heat generation is primarily provided by the oxidation of fatty acids derived from triglyceride hydrolysis and cellular uptake. Fatty acids also activate the uncoupling protein, UCP1, which creates a proton leak that uncouples mitochondrial oxidative phosphorylation from ATP production, resulting in energy dissipation as heat. Recent evidence supports the idea that in response to mild cold, ß-adrenergic signals stimulate not only lipolysis and fatty acid oxidation, but also act through the mTORC2-Akt signaling module to stimulate *de novo* lipogenesis. This opposing anabolic effect is thought to maintain lipid fuel stores during increased catabolism. We show here, using brown fat-specific Gs-alpha knockout mice and cultured adipocytes that, unlike mild cold, severe cold directly cools brown fat and bypasses ß-adrenergic signaling to inhibit mTORC2. This cell-autonomous effect both inhibits lipogenesis and augments UCP1 expression to enhance thermogenesis. These findings suggest a novel mechanism for overriding ß-adrenergic-stimulated anabolic activities while augmenting catabolic activities to resolve the homeostatic crisis presented by severe cold.

Brown and beige adipocytes convert oxidative energy to heat, largely by uncoupling cellular respiration from ATP production (1). This uncoupling is due predominantly to uncoupling protein-1 (UCP1), a mitochondrial transmembrane protein, which mediates a proton leak current that results in a heat producing futile cycle and acceleration of fatty acid oxidation (1, 2, 3, 4, 5). In humans, the energy expending activity of brown and beige adipocytes is inversely correlated with body fat, and these cell types play a key protective role in metabolic diseases such as diabetes and obesity (6). Thus, understanding the factors that control brown and beige fat metabolism is crucial for the development of treatments and preventive measures for metabolic disease, diabetes, and consequences of obesity (6, 7, 8).

Research into the signaling mechanisms that mediate the effects of ambient temperature on brown fat (brown adipose tissue; BAT) metabolism has focused primarily on the role of the sympathetic nervous system (SNS), which acts through catecholamine-activated ß-adrenergic receptors (predominantly ß3) to stimulate G-protein alpha-S (Gs-alpha; gene name, Gnas)-dependent activation of adenylyl cyclase, increase cellular cAMP levels, and activate protein kinase A (PKA) (9). It is well established that this signaling pathway can modulate multiple aspects of BAT metabolism including lipolysis and UCP1-dependent thermogenesis (1). It is also well established that BAT—like white adipose tissue—responds to anabolic signals (*e.g.*, insulin), which act through the mTORC2-Akt signaling module to phosphorylate and activate ATP-citrate lyase (ACLY), a key enzyme in *de novo* lipogenesis (DNL). More recently, it has been shown that chronic mild cold can act through ß3-adrenergic signaling in BAT to similarly activate mTORC2-Akt and stimulate DNL (10, 11), which prevents depletion of lipid stores in the face of increased lipolysis (10, 12, 13).

In contrast to mild cold, severe cold represents an acute homeostatic crisis, which we reasoned might evoke distinct BAT responses. In particular, we hypothesized that lipogenesis might be transiently silenced in response to severe cold as catabolic activities (*e.g.*, lipolysis and thermogenesis) are maximized. Based on a recent report that cold can have direct effects in adipocytes (14), we further hypothesized that differences between mild and severe cold might reflect cooling of BAT itself, coupled with divergence between adrenergic and local signals.

## Results

### Divergent effects of mild and severe cold on mTORC2 signaling in response to BAT temperature

**Figure 1:**
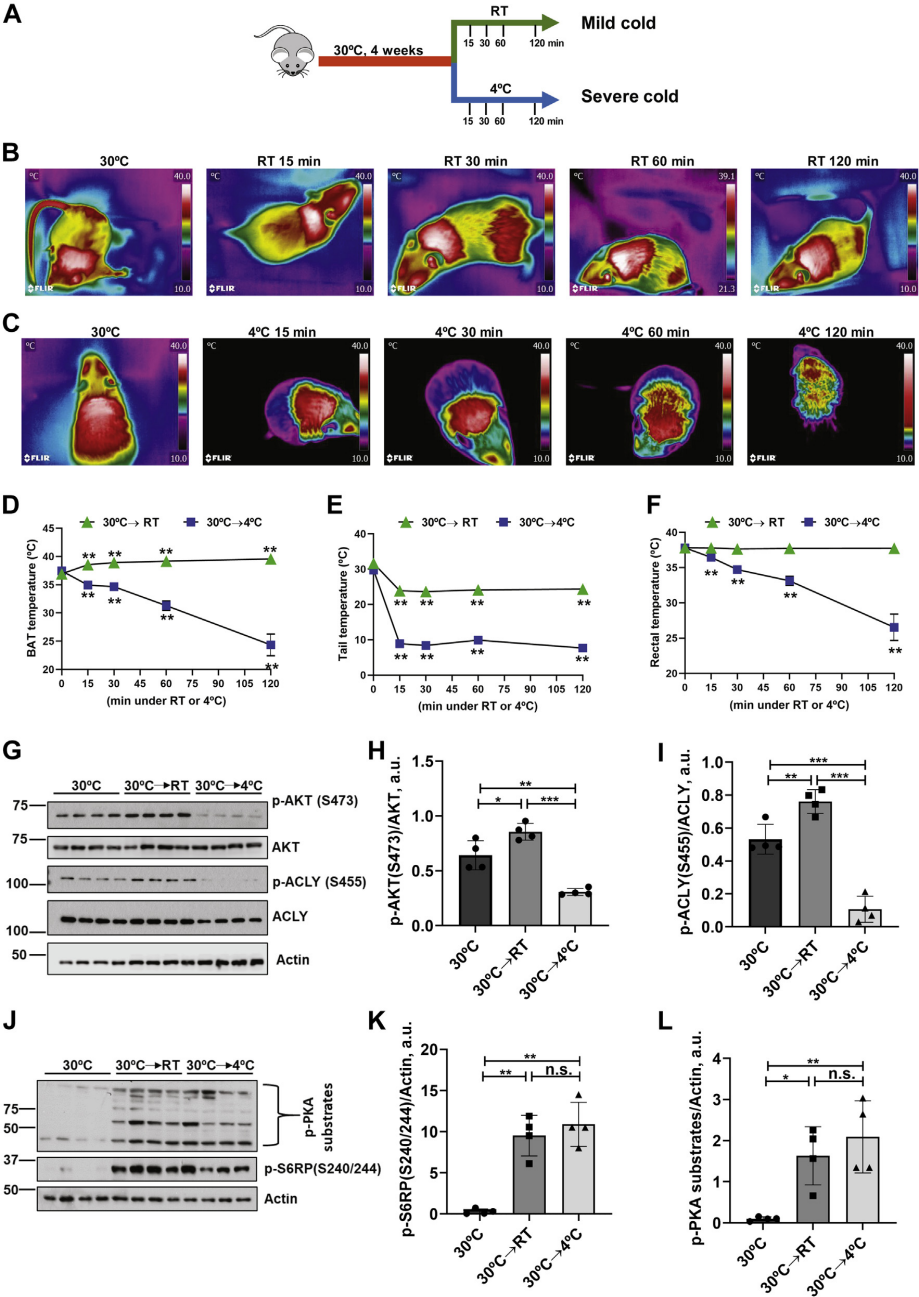
**Divergent effects of mild and severe cold on BAT temperature and mTORC2 signaling.***A*, schematic design of the experiment. Male wild-type C57B6 mice (n = 28) were acclimated to thermoneutrality (30 °C) for 4 weeks followed by exposure to either RT/mild cold (n = 14) or 4 °C/severe cold (n = 14) for various time points. *B* and *C*, infrared images of mice exposed to RT (*B*) or 4 °C (*C*) for times shown. Images are displayed using the rainbow high contrast color palette in the FLiR Research IR program using a temperature linear display between 10 and 40 °C. *D* and *E*, compiled infrared temperature data for BAT (*D*) and tail (*E*). *F*, rectal temperature (direct measurement with rectal probe). *G*, male wild-type C57B6 mice (n = 4/group) were acclimated to thermoneutrality (30 °C) followed by exposure to RT or 4 °C for 1 h iBAT was collected and subjected to immunoblot for Akt/pS473, total AKT, ACLY/pS455, total ACLY, and Actin. *H*, bar graph representing image quantification of Akt/pS473 normalized to total AKT. *I*, bar graph representing image quantification of ACLY/pS455 normalized to total ACLY. *J*, iBAT lysates immunoblotted for p-S6RP/S240/244 (mTORC1 substrate), p-PKA substrates, and Actin. *K*, bar graph representing image quantification of p-S6RP/S240/244 normalized to actin. *L*, image quantification of p-PKA substrates normalized to actin. Difference between groups was calculated by unpaired two-tailed *t*-test, ∗*p* < 0.05, ∗∗*p* < 0.01, and ∗∗∗*p* < 0.001.

As a first step in exploring the possibility of divergence, we compared the effects of acute mild and severe cold on iBAT (interscapular brown adipose tissue), tail, and rectal temperatures in C57bl/6j mice. Mice were acclimated to 30 °C and then transferred to either RT (23 °C) or 4 °C, and temperature was monitored over 2 h by infrared thermography (tail and iBAT) and direct measurement (rectum) (Fig. 1, *A*–*C*, Fig. S1, *A* and *B*). In response to mild cold exposure (from 30 °C to RT/23 °C), iBAT temperature rose slightly within 15 min, while tail temperature dropped, and rectal temperature remained constant (Fig. 1, *D–F*; green triangles). In contrast, in response to severe cold (from 30 °C to 4 °C exposure), iBAT, tail, and rectal temperatures all fell (Fig. 1, *D–F*; blue squares). In light of the recent evidence implicating mTORC2-Akt signaling in the anabolic effects of mild chronic cold (13), we next examined iBAT Akt/S473 phosphorylation (Akt/pS473) over the same interval as in Figure 1*B*. As shown in Figure S1, *C* and *F*, the responses to mild and severe cold were strikingly different: in mice exposed to mild cold (RT), Akt phosphorylation was slowly and monophasically stimulated, as was phosphorylation of ACLY (Fig. S1, *C–E*). Note that during this time, iBAT ß-adrenergic activity increased, as reflected by increased phosphorylation of PKA substrates, (Fig. S1, *I* and *J*), which likely accounts for the increase in iBAT temperature. In contrast, the iBAT response to severe cold was biphasic over time, with an initial rapid increase in Akt/pS473 and ACLY/pS455, followed by marked inhibition beginning at ∼30–60 min (Fig. S1, *F–H*). p-PKA substrate levels increased rapidly and remained elevated even as Akt/pS473 and ACLY/pS455 were inhibited (Fig. S1, *F*, *L*, and *M*). Direct comparison of mild and severe cold at a fixed time (1 h) further confirmed that the temperature responses of Akt/pS473 and ACLY/pS455 were biphasic, as well (Fig. 1, *G*–*I*). Note that cold also stimulated mTORC1 activity, consistent with a prior report (15); however, the effect was not biphasic (Fig. S1, *I* and *K*, Fig. S1, *L* and *N* and Fig. 1, *J* and *K*); both mild cold and severe cold had a stimulatory effect.

Cold stimulation also induces the hydrolysis of intracellular triglycerides stores in fat depots (lipolysis) (16). As a functional measures of lipolysis, we measured BAT triglycerides in cold-exposed mice. One hour of severe cold exposure induced lipolysis in BAT, manifested by the decrease of triglyceride levels in the tissue (Fig. S1*O*).

These data demonstrate that mild cold and severe cold evoke qualitatively distinct responses in mTORC2 activity: mild cold triggers a monotonic increase in mTORC2 activity (Fig. S1*D*), while severe cold invokes a biphasic response with initial increase in activity followed by a fall to baseline (Fig. S1*G*). It is also notable that the decrease in mTORC2 activity elicited by severe cold occurred in the face of sustained PKA activity and was preceded by a drop in temperature of brown fat itself, consistent with the idea that local temperature was directly responsible and was overriding ß-adrenergic signaling.

### Effects of mild cold—and early effects of severe cold—on mTORC2 signaling are abrogated in brown-fat-specific Gs-alpha knockout mice

**Figure 2:**
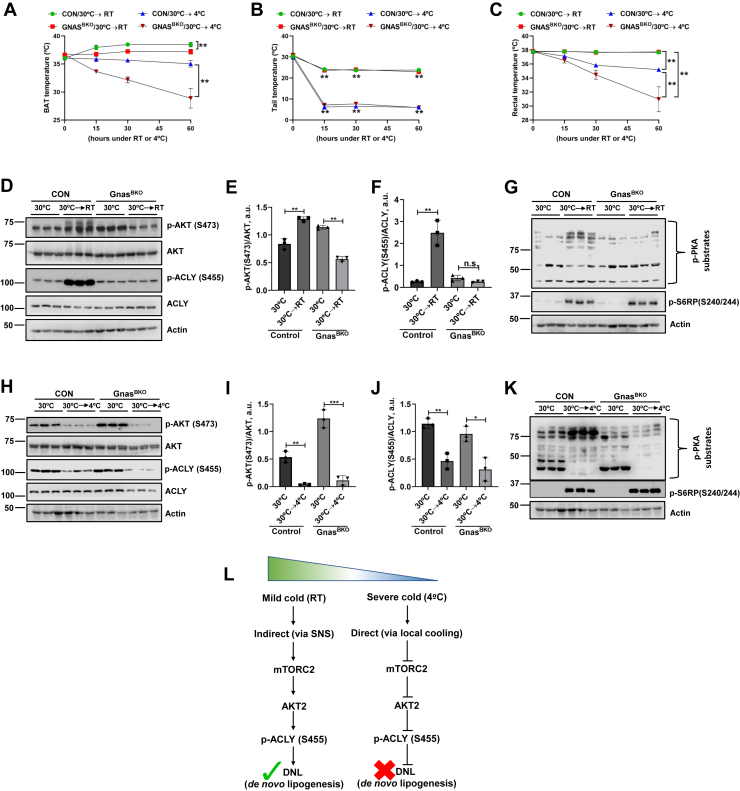
**Effects of mild but not severe cold on mTORC2-dependent signaling are negated in Gnas**^**BKO**^**(brown fat KO) mice**. Control mice (Gnas^f/f^; UCP1 Cre−) (n = 6) and Gnas^f/f^; UCP1 Cre + (n = 6, designated as Gnas^BKO^) were acclimated to thermoneutrality (30 °C) for 4 weeks. After acclimatization, Gnas^f/f^ controls (n = 3) and Gnas^BKO^ (n = 3) were exposed to RT or 4 °C for 1 h. *A* and *B*, compiled infrared temperature data measured at 15 min interval for BAT (*A*) and tail (*B*). *C*, Rectal temperature (measured with rectal probe). *D*, after acclimatization at thermoneutrality (30 °C) for 4 weeks, Gnas^f/f^ controls (n = 3) and Gnas^BKO^ mice (n = 3) were exposed to RT for 1 h, mice were euthanized, BAT was assayed by immunoblot for mTORC2 (Akt/pS473), total AKT, ACLY/pS455, total ACLY, and Actin. Bar graphs represent image quantification of Akt/pS473 normalized to total AKT (*E*) and ACLY/pS455 normalized to total ACLY (*F*). Immunoblot for PKA (phosphor-PKA substrates) activities (*G*). *H*, after acclimatization at thermoneutrality (30 °C) for 4 weeks, Gnas^f/f^ controls (n = 3) and Gnas^BKO^ mice (n = 3) were exposed to 4 °C for 1 h, mice were euthanized, BAT was assayed by immunoblot for mTORC2 (Akt/pS473), total AKT, ACLY/pS455, total ACLY, and Actin. Bar graphs represent image quantification of Akt/pS473 normalized to total AKT (*I*) and ACLY/pS455 normalized to total ACLY (*J*), immunoblot for PKA (phosphor-PKA substrates) activities (*K*). Statistical difference among controls 30 °C to or Gnas^BKO^ animals was calculated by unpaired two-tailed *t*-test. ∗*p* < 0.05, ∗∗*p* < 0.01, and ∗∗∗*p* < 0.001. *L*, flow diagram summarizing indirect (*via* SNS) and direct (*via* local cooling) effects of mild and severe cold on mTORC2-DNL regulation. Note: *panels H* and *K* are from the same gel; actin stain is shown in both for clarity of presentation.

In order to further explore the possibility of local temperature overriding ß-adrenergic signaling, we turned to genetically modified mice lacking ß-adrenergic signaling within BAT due to selective deletion of Gnas (encoding Gs-alpha). Gs-alpha acts through cAMP to activate PKA and Epac and is essential for all ß-adrenergic signaling in brown adipocytes (9, 10, 17). We first examined the effects of mild cold at 1 h by which time wild-type mice had manifested an increase in both pAkt and pACLY (Figs. 1*G* and S1). Stimulation by mild cold was completely lost in Gnas^BKO^ mice (Fig. 2, *D–F*), and in fact, Akt phosphorylation decreased significantly (Fig. 2*E*); pACLY also tended to decrease; however, this effect did not reach significance (Fig. 2*F*). Furthermore, the early stimulatory effect of severe cold on mTORC2 (15 min following transition from thermoneutrality) was also lost in Gnas^BKO^ mice (Fig. S1, *P* and *Q*), supporting the idea that the transient activation of mTORC2 by severe cold exposure is dependent on ß-adrenergic signaling (Fig. S1, *P* and *Q* *versus* Fig. S1, *F* and *G*). Importantly, the inhibitory effects of severe cold on pAkt and pACLY in Gnas^BKO^ were fully sustained and statistically indistinguishable from those in control mice (Fig. 2, *H–J*). As expected (9), responses of PKA to both mild and severe cold were lost in the Gnas^BKO^ mice (Fig. 2, *G* and *K*). We have also measured iBAT, tail, and rectal temperatures from Gnas^BKO^ mice (Fig. 2, *A*–*C*). Notably, in contrast to control mice, iBAT temperature failed to increase in Gnas^BKO^ mice exposed to RT, consistent with its dependence on ß-adrenergic signaling (Fig. 2*A*). In response to severe cold, the Gnas^BKO^ mice show much more dramatic drops in BAT and rectal temperatures than control mice (Fig. 2, *A* and *C*), which are likely due to loss of adrenergic signaling. Mice with genetic disruption of all three beta adrenergic receptors (β-less mice (18)) demonstrated similar results (Fig. S2).

### β(3)-adrenergic signaling does not inhibit, but rather, stimulates mTORC2 activity in iBAT

**Figure 3:**
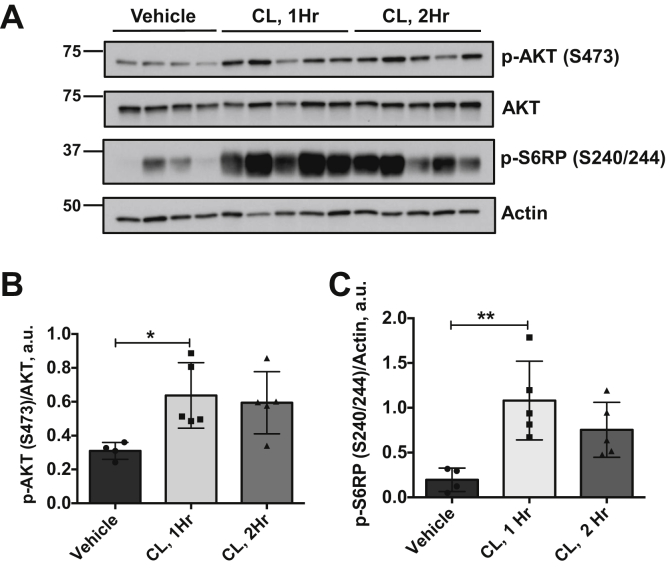
**The β(3)-adrenergic agonist CL 316,243 stimulates mTORC2.** Male mice (n = 14) were acclimated to thermoneutrality for 14 days followed by treatment with vehicle (n = 4) or CL 316,243 treatment for 1 h (n = 5) or 2 h (n = 5). iBAT was harvested and assayed by immunoblot to detect targets as shown (*A*). Bar graphs show image quantification of Akt/pS473 normalized to total AKT (*B*), p-S6RP/S240/244 normalized to actin (*C*). Difference between groups was calculated by one-way ANOVA followed by Tukey's multiple comparison post-hoc test. ∗*p* < 0.05 (F = 5.147, *p* = 0.026 for p-AKT graph) and ∗∗*p* < 0.01 (F = 8.033, *p* = 0.007 for p-S6RP graph).

In order to assess directly the effects of ß3-adrenergic receptor activation, we treated mice maintained at thermoneutrality with the selective ß(3)-agonist CL 316,243 and assessed iBAT mTORC2 activity, as above. Within 1 h of CL treatment, both mTORC1 and mTORC2 activities were significantly increased relative to vehicle-treated controls (Fig. 3), consistent with previous reports (10, 13, 15). This resembles the effect of mild cold and the early effect of severe cold, but is opposite to the later effect of severe cold. These results further suggest that the effect of mild cold is indirect (*via* SNS) and the effect of severe cold is direct (*via* local cooling) (Fig. 2*L*).

### mTOR inhibition causes elevated thermogenic gene expression in iBAT of mice maintained at thermoneutrality

**Figure 4:**
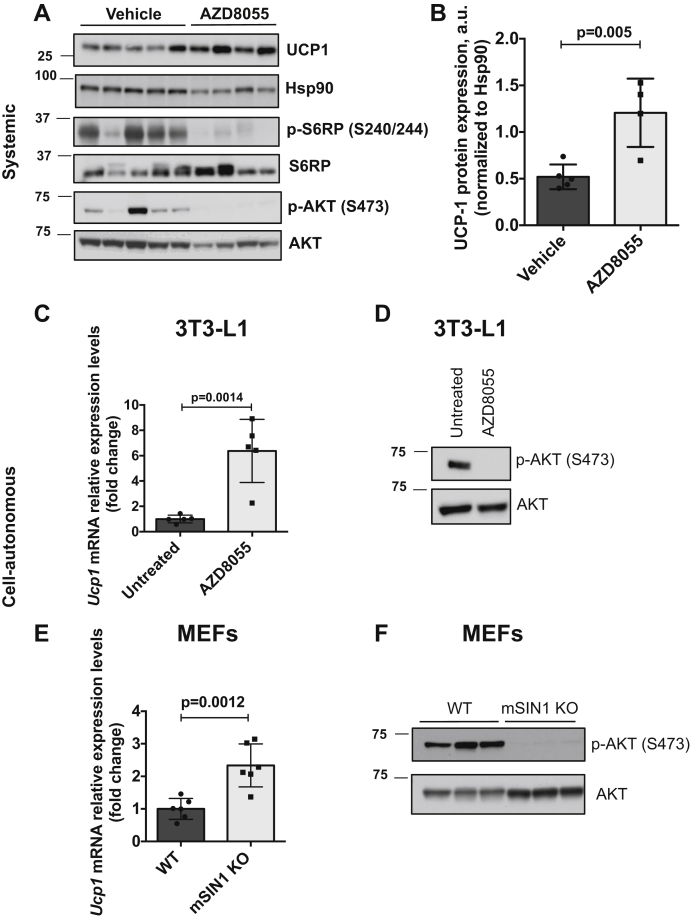
**Pharmacologic inhibition of mTOR stimulates UCP1 expression in mouse iBAT and in differentiated 3T3-L1 and MEFs.** Male mice (n = 5/vehicle group, for AZD8055 group n = 4) were acclimated to thermoneutrality for 2 weeks followed by treatment with either vehicle or AZD8055 (15 mg/kg/BW) for 20 h. Animals were sacrificed, and iBAT was isolated and assayed by western blot for thermogenic protein: UCP1, mTORC1, and two targets: S6RP/pS240/244 and Akt/pS473, S6RP and AKT (*A*). Bar graph represents image quantification of UCP1 immunoblot, Hsp90 was used to normalize UCP1. Difference between groups was calculated by unpaired two-tailed *t*-test (*B*). *C*, fully differentiated 3T3-L1 adipocytes were treated with AZD8055 (0.5 μM) for 4 h (n = 5) and total RNA was isolated and mRNA expression of UCP1 was analyzed by qPCR. TBP was used as normalizing control gene. Statistical difference between untreated and AZD8055 groups was calculated by unpaired two-tailed *t*-test. To test for the efficiency of AZD8055, whole-cell lysates were immunoblotted for Akt/pS473 and AKT (*D*). *E* and *F*, genetic deletion of mTORC2. WT and mSin1 knockout (mSIN1 KO) murine embryo fibroblasts (MEFs) were differentiated into adipocytes and assayed for UCP1 mRNA expression by qPCR (n = 6). Statistical difference between WT and mSIN1 KO groups was calculated by unpaired two-tailed *t*-test (*E*). Genetic deletion/loss of mTORC2 function was confirmed by probing whole-cell lysates for Akt/pS473 and AKT (*F*).

In order to further explore the role of mTOR in mediating the effects of cold, we examined the effects of pharmacologic inhibition of mTOR in mice maintained at thermoneutrality. Following 2 weeks adaptation to 30 °C, mice were treated with either AZD8055 (a potent inhibitor of both mTORC1 and mTORC2), or vehicle for 20 h, sacrificed, and whole iBAT tissue was prepared for immunoblot. As shown in Figure 4, mTOR inhibition by AZD8055 caused a significant increase in UCP1 protein expression (Fig. 4, *A* and *B*) compared with vehicle-treated mice. As expected, AZD8055 abolished Akt phosphorylation (Fig. 4*A*). Thus, pharmacologic inhibition of mTOR increases thermogenic gene expression selectively in mouse iBAT.

### Lowering ambient temperatures directly inhibits mTORC2 activity in cultured adipocytes

**Figure 5:**
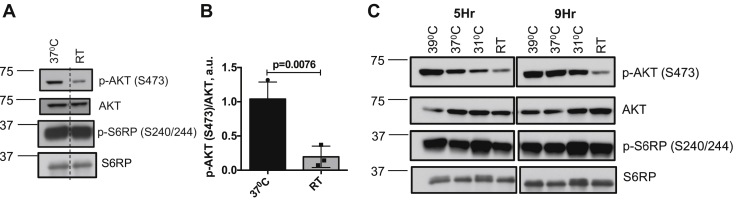
**Cooler temperatures inhibit mTORC2 activity in differentiated 3T3-L1 adipocytes.** Differentiated 3T3-L1 adipocytes, acclimated to 37 °C, were exposed to room temperature (24.5 °C) for 4 h, and whole-cell lysates were analyzed by western blot (*A*) to assess expression levels of Akt/pS473, AKT, p-S6RP/S240/244, and S6RP. *Dashed line* represents image splicing to remove irrelevant data in gel. *B*, bar graph representing average image quantification of Akt/pS473 normalized to total AKT from three separate experiments. Statistical difference between 37 °C and RT groups was calculated by unpaired two-tailed *t*-test. *C*, differentiated 3T3-L1 adipocytes were acclimated to 37 °C and then shifted to 39 °C, 37 °C, 31 °C, or RT (23 °C) for 5 or 9 h and whole-cell lysates were probed for Akt/pS473, AKT, p-S6RP/S240/244, and S6RP. Note progressive decrease in Akt/pS473 levels with decreasing temperature, in contrast to biphasic effect in mouse iBAT.

The foregoing data establish that severe cold does not act through the canonical ß-adrenergic signaling pathway to inhibit mTORC2 in mice. Moreover, the kinetics of change in iBAT temperature relative to Akt/pS473 level following exposure to severe cold are consistent with integration of early ß-adrenergic effects with slower direct effects of cold within iBAT itself (compare Fig. 1*D*, blue squares and Fig. S1*F*). However, whole animal experiments cannot rule out all potential systemic influences. We therefore examined responses to cold of two different adipocyte models, differentiated LgT (19) and 3T3-L1 cells (20). In 3T3-L1 cells, which have mixed WAT/BAT characteristics (20), lowering ambient temperature from 37 °C to 24.5 °C reduced Akt/pS473 to 18.8% of its baseline level (Fig. 5, *A* and *B*). Cool temperature had a similar effect on a distinct mTORC2 target, serum, and glucocorticoid regulated kinase-1 (SGK1; Fig. S3). On the other hand, lowering ambient temperature did not similarly inhibit mTORC1 activity (Fig. 5*C*). Importantly, IRS-1 Tyr phosphorylation (a reflection of insulin receptor activation) was not reduced in the low-temperature condition and was in fact elevated (Fig. S4), suggesting that upstream insulin signaling was intact, consistent with the findings of Gasparetti *et al.* (21). In a temperature titration experiment, (Fig. 5*C*), Akt/pS473 progressively decreased as temperature was lowered from 37 °C to 31 °C or RT and increased in response to shift to 39 °C. We also observed that Akt/pS473 was markedly decreased in 3T3-L1 cells exposed to severe cold (4 °C) compared with cells maintained at 37 °C (data not shown). We also performed similar experiments on LgT cells (immortalized mouse brown adipocytes) and found similarly that Akt/pS473 was progressively reduced in response to lowering temperature (Fig. S5). These effects in cultured adipocytes are in marked contrast to the biphasic response of iBAT to severe cold in wild-type mice, but resembled the iBAT response in Gnas^BKO^ and ß-less mice. It is also notable that the effect of ß-adrenergic stimulation in wild-type mice (Fig. 3) resembled the effect of mild cold (Fig. 1*G*). Furthermore, the effect of cold in cultured adipocytes was not biphasic (increasing cold had a monophasic inhibitory effect on mTORC2) and resembled the effect of severe cold in mice (Fig. 1*G*).

### Cool temperatures or pharmacologic or genetic deletion of mTORC2 induces thermogenic gene expression in cultured adipocytes

**Figure 6:**
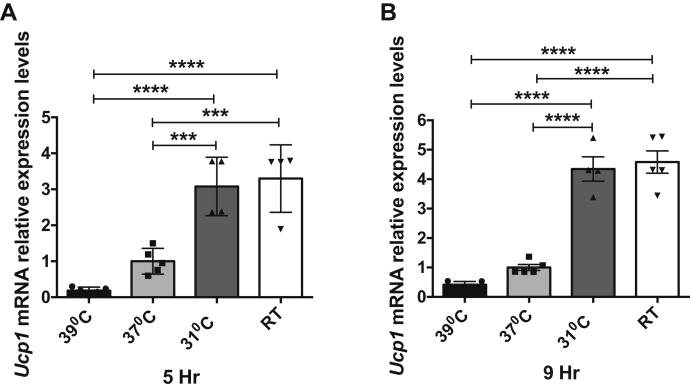
**Cool temperatures induce thermogenic gene expression in cultured 3T3-L1 adipocytes.** Fully differentiated 3T3-L1 adipocytes were exposed to 39 °C, 37 °C, 31 °C, or RT (23 °C) for 5 or 9 h. Total RNA was isolated and mRNA expression of UCP1 was analyzed by qPCR. TBP was used for internal normalization. Values shown are normalized to cells exposed to 37 °C (n = 4–5 in each group). Difference between groups was calculated by one-way ANOVA followed by Tukey's multiple comparison post-hoc test. ∗∗∗*p* < 0.001, ∗∗∗∗*p* < 0.0001. For UCP1, 5 h graph (*A*) F = 28.69, *p* < 0.0001; UCP1, 9 h graph (*B*) F = 65.79, *p* < 0.0001.

We next examined *UCP1* expression over the same temperature range associated with mTORC2 inhibition. In cells acclimated to 37 °C, lowering medium temperature to 31 °C or RT significantly increased *UCP1* expression, whereas raising temperature tended to suppress *UCP1* below baseline (Fig. 6). These findings are consistent with those of Ye *et al*. (14), who demonstrated that physiologically relevant temperature reductions stimulate expression of key thermogenic genes in cultured adipocytes in the absence of any change in ß-adrenergic/cAMP-dependent signaling. Finally, we found that pharmacologic inhibition (Fig. 4, *C* and *D*) or genetic ablation (using CRISPR-mediated deletion of mSin1; Fig. 4, *E* and *F*) of mTORC2 stimulated *UCP1*, consistent with the *in vivo* data (Fig. 4, *A* and *B*).

## Discussion

**Figure 7:**
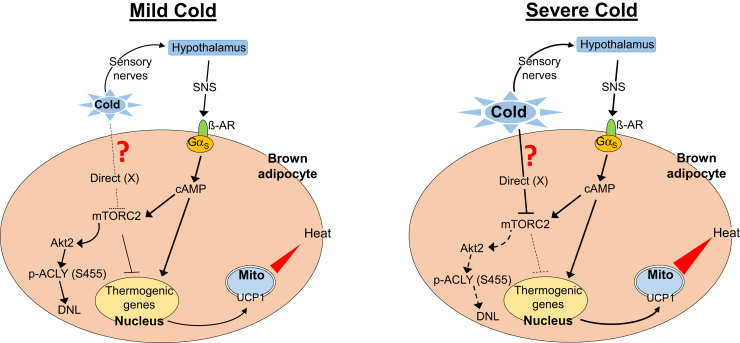
**Schematic diagram showing adipocyte integrated responses to cold.** Shown are responses to mild (*left panel*) *versus* severe (*right panel*) cold. Mild cold acts through the SNS to stimulate ß-adrenergic signaling, increase intracellular cAMP, and stimulate UCP1 expression. At the same time, cAMP stimulates mTORC2-dependent phosphorylation of Akt at S473, which stimulates lipogenesis *via* p-ACLY (S455), while blunting the stimulation of UCP1 expression. The net result is moderate stimulation of UCP1 together with enhanced DNL. In contrast, in addition to the canonical effect through the SNS, severe cold has a cell-autonomous PKA-independent effect to inhibit mTORC2 through an unknown cold receptor/sensor (referred to here as “?”/“X”). Stimulation of the thermogenic program is augmented as the inhibitory effect of mTORC2 is lifted. At the same time mTORC2 inhibition leads to a decrease in active Akt (pS473), and thus the SNS-dependent stimulation of lipogenesis through activation of Akt and p-ACLY (S455) is blocked. See text for further details.

Our present data identify a novel cell-autonomous pathway for regulation of brown fat metabolism, the effects of which are integrated with adrenergic signaling to produce a biphasic response to cold, as shown schematically in Figure 7. According to this view, in the presence of mild cold, as the skin—but not deeper tissue—is cooled, ß-adrenergic signaling predominates, acting through canonical cAMP-dependent signaling to stimulate both catabolic and anabolic processes: lipolysis and fatty acid oxidation are increased, and UCP1-dependent thermogenesis is stimulated. But, ACLY-dependent DNL is also enhanced (Fig. 7, left panel), as has been described (13); a balance between DNL and lipolysis is established and lipid stores are sustained. In contrast, in response to severe cold, as BAT itself cools, the mTORC2-Akt-ACLY signaling module is inhibited and DNL is reduced (Fig. 7, right panel). mTORC2-dependent blunting of lipolysis and UCP1 expression is also relieved, thus augmenting ß-adrenergic stimulation of thermogenesis. Consistent with this idea, in mice exposed to mild cold, mTORC2-dependent phosphorylation of Akt and ACLY was slowly stimulated (in parallel with iBAT ß-adrenergic activity, as reflected by increased phosphorylation of PKA substrates). In contrast, iBAT mTORC2 was initially stimulated in mice exposed to severe cold, reflecting ß-adrenergic stimulation as superficial tissues cooled. But then as iBAT itself cooled, mTORC2 was markedly inhibited beginning at ∼30–60 min ß-adrenergic activity did not follow this biphasic response, but rather increased rapidly and remained elevated even as mTORC2 was inhibited (Fig. 1). This biphasic temperature response was lost in Gnas^BKO^ mice (lacking Gs-alpha, Fig. 2). Strikingly, mTORC2 was progressively inhibited by both mild and severe cold (Figs. 2 and S1).

These signaling changes were accompanied by concordant changes in iBAT *UCP1* levels, which were substantially greater in animals exposed to severe cold than in those exposed to mild cold (Fig. S6), a result that is consistent with prior work showing that genetic disruption of mTORC2 (by knocking out Rictor) enhances the UCP1 response to cold (22). Another functional manifestation of distinct local and ß-adrenergic effects is reflected by the observation that iBAT TG levels were decreased significantly in animals exposed to severe but not to mild cold (Fig. S1*O*). Finally, the temperature response of iBAT was also consistent with the convergence of ß-adrenergic and local signals (Figs. 1, 2 and S1).

A key area for future investigation is the identification and characterization of the adipocyte sensor for these direct cold effects. Plausible candidates include members of the Trp temperature-sensitive receptor family (23). In particular TrpV1, TrpV2, TrpV4, and TrpM8 are expressed in brown adipocytes and have been found to play roles in adipose tissue differentiation and metabolism (23, 24, 25). In this regard, we measured BAT mRNA levels of TRPV1, TRPV2, TRPV3, TRPV4, and TRPM8, channels in mice exposed to 4 °C for 4 h, and found that TRPV3 and TRPV4 were significantly upregulated, paralleling *UCP*-1 (Fig. S6), consistent with a role in responding to severe cold, and invoking a rescue mechanism to shift metabolic activity away from DNL and entirely toward thermogenesis. Another appealing (not mutually exclusive) possibility is that mTORC2 itself is directly modulated by cold. In this regard, it is notable that we (Figs. 5 and S5) and others (26) have demonstrated specific cold-induced inhibition of mTORC2 in multiple cell types, under conditions in which mTORC1 is unaffected.

Thus, our present findings identify for the first time a rescue pathway, which signals through cell-autonomous regulation of the mTORC2-Akt kinase cascade to both override adrenergic stimulation of DNL and synergistically stimulate Ucp1. We propose that this direct effect, which leads to transient suppression of anabolic and enhanced catabolic (thermogenic) activity, contributes to resolving the homeostatic crisis presented by exposure to severe cold. Further work is needed to fully define the sensors that mediate these cell-autonomous effects and to fully explore the physiological parameters of the response. In light of the disappointing results with ß3-adrenergic agonists for the treatment of obesity, the identification of local cold effects on lipogenesis, which counter anabolic while enhancing catabolic ß-adrenergic effects, suggests new avenues to explore in the battle against metabolic disease.

## Experimental procedures

### Animal experiments

Male C57BL/6 mice (JAX #000664) and Ucp1-Cre (JAX #024670) were purchased from the Jackson Laboratory. Gnasf/f mice in 129S6/SvEvTac Black Swiss background were provided by Dr Lee S Weinstein (27). The βless mice were provided by Dr Shingo Kajimura. Experiments were carried out in mice that were 10–14 weeks of age.

### Acute mild and severe cold challenge experiments

Ten-week-old male mice (n = 28) were acclimated to thermoneutrality (30 °C) for 4 weeks and then single with free access to food and water. The core body temperature prior and during mild cold (RT) or severe cold (4 °C) exposure was monitored using BAT-12 Microprobe Thermometer with probe RET-3 (Physitemp) at the indicated times (15, 30, 60, and 120 min). BAT and tail temperature was recorded with a thermal imaging camera (FLIR i3, FLIR Systems) and analyzed with FLIR QuickReport software. After temperature measurement, mice were euthanized, and iBAT was assayed for Akt/pS473, AKT, ACLY/pS455, ACLY, S6RP/p(S240/244), and phosphor-PKA substrates. In another set of experiment, male mice (n = 12) were acclimated to thermoneutrality (30 °C) for 2–4 weeks. For acute mild cold challenge, mice (n = 4) and for acute severe cold challenge, mice (n = 4) were housed individually, placed at either RT or 4 °C for 1 h, with free access to food and water. Control mice (n = 4) were maintained at thermoneutrality. Mice were euthanized and iBAT was assayed to immunoblot for Akt/pS473, AKT, ACLY/pS455, ACLY, S6RP/p(S240/244), and phosphor-PKA substrates.

### Selective β3-agonist experiments

Male mice (n = 14) were acclimated to thermoneutrality (30 °C) for 14 days. After acclimatization, mice were injected intraperitoneally either with vehicle or CL 316,243, a selective β3-agonist (1 mg/kg/BW) (Sigma-Aldrich) and placed back at thermoneutrality. One to two hours after CL 316,243 administration, mice were euthanized, and iBAT was dissected and frozen immediately in liquid nitrogen. iBAT tissue lysates were used to detect Akt/pS473, AKT, and p-S6RP/S240/244.

### Gnas^BKO^ experiments

Gnas^f/f^ male mice (n = 12) (Controls) and Gnas^BKO^ male mice (n = 12) were acclimated to thermoneutrality (30 °C) for 4 weeks. After acclimatization, Gnas^f/f^ mice (n = 3) and Gnas^BKO^ mice (n = 3) were exposed to either mild cold (RT) or severe cold (4 °C) for 1 h (housed individually and placed at RT or 4 °C). Controls for the experiment: Gnas^f/f^ mice (n = 3) and Gnas^BKO^ mice (n = 3) were kept at thermoneutrality. BAT, tail, and rectal temperatures were measured at 15 min interval as described above. After 1 h of acute mild or severe cold challenge, mice were euthanized and iBAT lysates were prepared for immunoblot and assayed for Akt/pS473, AKT, ACLY/pS455, ACLY, and phosphor-PKA substrates. For early response to severe cold, GnasBKO male mice (n = 3) were acclimated to thermoneutrality (30 °C) for 4 weeks and then were exposed to severe cold (4 °C) for 15 min, BAT was collected and assayed for Akt/pS473, AKT, and phosphor-PKA substrates as described previously.

### β-less mice experiments

Control male mice (n = 6) and β-less male mice (n = 6) were maintained at thermoneutrality for 4 weeks. Three control mice and β-less mice (n = 3) were exposed to severe cold (4 °C) for 1 h. After 1 h of cold exposure, mice were euthanized and iBAT lysates were used to probe for Akt/pS473, AKT, ACLY/pS455, ACLY, and phosphor-PKA substrates.

### mTOR inhibitor experiments

Male mice (n = 9) were acclimated to thermoneutrality (30 °C) for 14 days. After acclimatization, vehicle (n = 5) or AZD8055 (n = 4) solution was injected intraperitoneally (i.p). AZD8055 solution was prepared by dissolving 15 mg/kg/BW AZD8055 powder (S1555, SelleckChem) in vehicle solution. The vehicle solution contained sterile saline with 20% DMSO and 40% polyethylene glycol (PEG). Mice were transferred back to the thermoneutral condition for 20 h. After 20 h of i.p. injection, mice were euthanized and iBAT was dissected and frozen immediately in liquid nitrogen. Western blot analysis was performed from the iBAT lysates to detect thermogenic protein:UCP1. To confirm mTOR inhibition by these inhibitors, we also immunoblotted with p-S6RP/S240/244 and Akt/pS473 antibodies.

### Adipocyte cell culture experiments

#### 3T3-L1 adipocytes

3T3-L1 mouse embryonic fibroblasts were obtained from ATCC (catalog no. CL-173), maintained, and chemically induced to differentiate them to adipocytes (according to the ATCC differentiation protocol). Cells were differentiated in six-well plates at 37 °C. For temperature shift experiments, differentiated 3T3-L1 cells were transferred to incubators maintained at 39 °C, 37 °C, 31 °C, or 24.5 °C. After exposing cells at these temperatures, cells are lysed and whole-cell extracts were used to immunoblot against p-S6RP/S240/244 and Akt/pS473. From the same experiment, RNA was isolated and qPCR was done to check *UCP1* gene expression levels. In a separate set of experiments, differentiated adipocytes were treated with 0.5 μM AZD8055 for 4 h and *UCP1* gene expression is assessed using qPCR, according to the manufacturer's guidelines, as described below. From the same experiment, whole-cell extracts were used to probe for Akt/pS473 and AKT protein levels. In parallel, lysates from cells maintained at 24.5 °C were immunoprecipitated with anti-phospho-tyrosine antibody and immunoblotted with anti-IRS1. In another experiment, differentiated 3T3-L1 adipocytes were treated with dexamethasone for 4 h to increase SGK1 expression and then either exposed to room temperature (24.5 °C) or maintained at 37 °C for 4 h, whole-cell extracts were used to probe for p-SGK1 (S422).

#### LgT (SV40T antigen-immortalized mouse brown preadipocytes) cells

LgT cells were cultured, maintained, and differentiated to mouse brown adipocytes as described (19). Temperature titration experiment was performed on these differentiated adipocytes, as described above for 3T3-L1 adipocytes.

#### Gene expression analysis by qPCR

Total RNA from cultured 3T3-L1 adipocytes or iBAT was isolated using Qiazol (Qiagen) method combined with Qiagen RNAEasy minicolumns, according to the manufacture's instructions. Equal amounts of RNA were retro-transcribed to cDNA using cDNA synthesis kit (Bio-Rad). The resultant cDNA was used in quantitative PCR reactions containing SYBR-green fluorescent dye (Bio-Rad). *UCP*1 relative expression levels were calculated using the 2^−ΔCt^ method. TATA-binding protein (TBP) or 36B4 expression was used for normalization. Target primer sequences are shown in Table 1.

**Table 1:** RT-PCR primer sequences

Gene name	Primer (5′−3′)	Primer (5′−3′)
*UCP1*	AAGCTGTGCGATGTCCATGT	AAGCCACAAACCCTTTGAAAA
*Tbp*	CCCTATCACTCCTGCCACACCAGC	GTGCAATGGTCTTTAGGTCAAGTTTAC
*UCP1*^a^	CGTACCAAGCTGTGCGATGT	ACCCGAGTCGCAGAAAAGAA
*36b4*^a^	TTTGGGCATCACCACGAAAA	GGACACCCTCCAGAAAGCGA
*TRPM8*^a^	ACAGACGTGTCCTACAGTGAC	GCTCTGGGCATAACCACACTT
*Trpv1*^a^	CCGGCTTTTTGGGAAGGGT	GAGACAGGTAGGTCCATCCAC
*Trpv2*^a^	TGCTGAGGTGAACAAAGGAAAG	TCAAACCGATTTGGGTCCTGT
*Trpv3*^a^	ACGGTCACCAAGACCTCTC	GACTGTTGGGATTGGATGGGG
*Trpv4*^a^	ATGGCAGATCCTGGTGATGG	GGAACTTCATACGCAGGTTTGG

a Primers used to do RT-PCR from brown fat.

#### Western blotting

Cells were lysed in lysis buffer containing 40 mM HEPES (pH 7.5), 120 mM NaCl, 50 mM NaF, 10 mM Sodium Pyrophosphate, 10 mM glycerophosphate, 1 mM EDTA (pH 8), 1% Triton-X-100 with complete protease inhibitor cocktail and PhoSTOP phosphatase inhibitors. Tissues were disrupted using tissue lyser II (Qiagen) and lysed in buffer containing 150 mM NaCl, 50 mM Tris-base, 1% NP-40, 0.1% SDS, 0.5% Sodium Deoxycholic acid (pH of lysis buffer is adjusted to 7.5) with complete protease inhibitor cocktail and PhoSTOP phosphatase inhibitors. Total protein from both cell and tissue lysates was estimated by Bradford assay. In total, 15–20 μg of total protein was run on SDS-PAGE, transferred to PVDF membrane, and probed with antibodies with specific primary antibodies. Antibodies used were UCP1 (CST, #14670), phospho- and total AKT (CST, #4060S and #9272S, respectively), phospho-S6RP (CST, #2215S), total S6RP (CST, #2217), phosphor-PKA substrate (CST, #9624), Phospho-ATP-Citrate Lyase (Ser455) (CST, #4331), ATP-Citrate Lyase (CST, #4332), and Hsp-90 (CST, #4877S) were purchased from Cell Signaling Technologies. p-SGK S422 (catalog no. SC-16745-R), p-Tyr (pY99) (catalog no. SC-7020), and Actin (catalog no. SC-1615) antibodies were purchased from Santa Cruz Biotechnology. Anti-IRS1 (catalog no. 06-248) was purchased from Millipore. Secondary antibodies conjugated with HRP were used for specific protein detection. Band intensities were quantified using NIH ImageJ software.

#### Lipolysis

Lipolysis *ex vivo* was performed as previously described (9). Briefly, tissue samples (∼20 mg) of interscapular brown fat pads from overnight fasted mice were dissected and incubated at 37 °C without shaking in 500 μl of KRB buffer (12 mM HEPES, 121 mM NaCl, 4.9 mM KCl, 1.2 mM MgSO_4_, and 0.33 mM CaCl_2_) containing 2% fatty acid free BSA and 0.1% glucose before and after 1 hour of acute mild (RT) or severe cold (4 °C) exposure. The KRB buffer was collected and triglycerides were measured using Free Glycerol Reagent (Sigma, F6428). The levels of free glycerol were normalized to the mass of the tissue sample.

#### MEFs

Murine embryo fibroblasts (MEFs), homozygous null for mSin1, one of the core components of mTORC2 (a kind gift of Bing Su (28)), were maintained and differentiated into adipocytes as described previously (29).

### Statistics

All data are presented as mean ± SD. Comparisons between more than two groups were assessed by one-way ANOVA followed by Tukey's multiple comparison post hoc test. Comparisons between two groups were assessed by unpaired Student's *t*-test (two-tailed). All the statistics were performed using GraphPad Prism version 6. *p* < 0.05 is considered as statistically significant value.

### Study approval

All animal studies described in this paper were conducted in accordance with all relevant guidelines and regulations, approved by the UCSF Institutional Animal Care and Use Committee (UCSF IACUC).

## Data availability

Data and materials reported in this study will be made publicly available by corresponding author.

## Supporting information

This article contains supporting information.

## Conflict of interest

The authors declare that they have no conflicts of interest with the contents of this article.
