# Supplementary material for: Role of mTORC2 in biphasic regulation of brown fat metabolism in response to mild and severe cold
Source: J Biol Chem. 2021 Apr 15;296:100632. doi: 10.1016/j.jbc.2021.100632 (PMC8121962; doi:10.1016/j.jbc.2021.100632)
Supplement: Figures S1 to S6 [file mmc1.pdf]

SUPPORTING INFORMATION

Role of mTORC2 in biphasic regulation of brown fat metabolism in response to mild and severe cold

Prasanna K. R. Allu[1, 4*#], Esther Paulo[2#], Ambre M. Bertholet[3], Gavin Situ[1, 6], Seung-Hwan Lee [2, 5], Yixuan Wu[2], Catherine E. Gleason[1, 6], Bidisha Saha[1], Ajay Chawla[2], Biao Wang[2] and David Pearce[1, 2*]

University of California at San Francisco, San Francisco, CA, USA, [1]Department of Medicine, Division of Nephrology. [2]Cardiovascular Research Institute. [3]Department of Physiology.
[4]Current location: Joslin Diabetes Center, Harvard Medical School, Boston, MA, 02215, USA.
[5]Current location: Division of Endocrinology and Metabolism, Department of Internal Medicine, Seoul St. Mary's Hospital, College of Medicine, The Catholic University of Korea, Seoul 06591, Korea.
[6]Current location: Circle Pharma, South San Francisco, CA, 94080, USA.

# Contributed equally.

* Co-corresponding authors:
Prasanna K. R. Allu
Section on Integrative Physiology and Metabolism
Joslin Diabetes Center
Harvard Medical School
Boston, MA, 02215
Ph: 628-224-7537
Prasanna.allu@joslin.harvard.edu

David Pearce
Mission Bay, Genentech Hall, Rm N272C
600 16th St.
San Francisco, CA 94158
Ph: 415-476-7015
Fax: 415-502-8644
david.pearce@ucsf.edu

The authors have no conflicts of interest to report.

Key words: mTOR, UCP1, cold, thermogenesis, lipogenesis, gene expression, brown adipose tissue, adipocytes.

Running title: Cold regulation of thermogenic fat.

SUPPLEMENTARY FIGURE LEGENDS:

Fig. S1. Effects of mild and severe cold on mTORC2 activity in iBAT . Male wild type C57B6 mice (n=28) were acclimated to thermoneutrality (30 °C) for 4-weeks followed by exposure to either RT (n=14) or 4 °C (n=14) for various time poitns. A different set of infrared images of mice exposed to 23^0C **(A)** or 4^0C **(B)** for times shown. Images are displayed using the rainbow high contrast color palette in the FLiR Research IR program using a temperature linear display between 10°C and 40°C. **C**. Immunoblots showing acute responses of mild cold (RT) on phosphorylation of Akt/s473 and its substrate, lipogenic enzyme, ACLY. Note monophasic increase in both Akt/pS473 and ACLY/pS455. Bar graphs represent image quantification of Akt/pS473 normalized to total AKT **(D)** and ACLY/pS455 normalized to total ACLY **(E). F.** Immunoblots showing acute responses of severe cold (4^0C) on Akt/pS473 and ACLY/pS455. Bar graphs represent image quantification of Akt/pS473 normalized to total AKT **(G)** and ACLY/pS455 normalized to total ACLY **(H).** Note biphasic effect. **I-K:** PKA (phosphor-PKA substrates) activities and mTORC1 (p-S6RP/S240/244) activities at RT. **L-N:** PKA (phosphor-PKA substrates) and p-S6RP/S240/24 at 4^0C. **O**: BAT triglycerides are calculated in the media from BAT in response to mild (RT) or severe cold (4^0C) (n=6). **P**. Gnas BKO mice (n=5) were acclimated to thermoneutrality for 4 weeks. After acclimatization, $Gnas^{BKO}$ (n=3) were exposed to 4 °C for 15 min. iBAT was assayed by western blot for Akt/pS473, total AKT, S6RP/p240-p244, total S6RP, PKA (phosphor-PKA substrates) and HSP90. Bar graphs represent image quantification of Akt/pS473 normalized to total AKT **(Q)** and S6RP/p240-p244normalized to total S6RP (**R**). Statisitical difference between groups was calculated by unpaired 2-tailed T-test, n.s. non significant, *** $p<0.001$.

Fig. S2. Effect of severe cold on mTORC2 and DNL is independent of all three β-adrenergic receptors signaling. Control mice (littermate controls) (n=6) and β-less mice (n=6) were acclimated to thermoneutrality (30 °C) for 4 weeks. After acclimatization at thermoneutrality (30 °C) for 4 weeks, controls (n=3) and β-less mice (n=3) were exposed to 4 °C for 1 hour, mice were euthanized, iBAT was assayed by western blot for mTORC2 (Akt/pS473), total AKT, ACLY/pS455, total ACLY, and Actin **(A)**. Bar graphs represent image quantification of Akt/pS473 normalized to total AKT **(B)** and ACLY/pS455 normalized to total ACLY **(C)**, Immunoblot for PKA (phosphor-PKA substrates) activities **(D)**. Bar graphs represent image quantification of PKA normalized to Actin **(E)** and pS6RP normalized to Actin **(F)**, Statistical difference among controls (30^0C vs. 30^0C to 4^0C) or β-less animals (30^0C vs. 30^0C to 4^0C) was calculated by unpaired 2-tailed T-test. *$p< 0.05$ and **$p< 0.01$. Note: Figs. S2A and S2D are from the same gel; actin stain is shown in both for clarity of presentation.

Fig. S3. Cold inhibits SGK1 phosphorylation in differentiated 3T3-L1 adipocytes. Differentiated 3T3-L1 adipocytes were treated with dexamethasone for 4 hours to increase SGK1 expression and then either exposed to room temperature (24.5 °C) or maintained at 37 °C for 4 hours. (**A**) Whole cell lysates were prepared and analyzed by western blot and expression levels of p-SGK (S422) and Tubulin were quantitated by densitometry. (**B**) Bar graph represents image quantitation of p-SGK (S422) normalized to tubulin. Statistical difference between 37 °C and RT groups was calculated by unpaired 2-tailed T-test.

Fig. S4. IRS-1 phosphorylation is intact in cold exposed differentiated 3T3-L1 adipocytes. Differentiated 3T3-L1 adipocytes were exposed to room temperature (24.5 °C) for 4 hours, and cell lysates were immunoprecipitated with anti-pY antibody and then immunoblotted with anti-IRS-1 antibody to detect p-Tyr IRS1 levels. (**A**). Whole cell lysates were also analyzed by western blot to detect IRS-1 expression levels. (**B**). Bar graph represents image quantitation of p-Tyr IRS-1 normalized to IRS-1 Statistical difference between 37 °C and RT groups was calculated by unpaired 2-tailed T-test.

Fig. S5 Lowering temperature inhibit mTORC2 activity in differentiated LgT brown adipocytes. Differentiated LgT adipocytes were adapted to 37°C and then exposed to various temperatures for 5 hours, as shown. Whole cell lysates were immunoblotted for p-AKT (S473), AKT, p-S6RP (S240/244), S6RP and Tubulin. Note that, p-AKT (S473) levels were decreased in a graded fashion at lower temperatures similar to 3T3-L1 adipocytes **(A)**. Bar graph represent image quantitation of p-AKT (S473) normalized to AKT. Difference between groups was calculated by one-way ANOVA followed by Tukey's multiple comparison post-hoc test. * $p<0.05$, ** $p<0.01$. $F=39.69$, $p=0.002$ **(B)**.

Fig. S6. Effects of mild cold vs. severe cold on iBAT thermogenic gene expression. UCP1, TRPM8, Trpv1, Trpv2, Trpv3 and Trpv4 gene expression was analyzed in iBAT of mice acclimated to thermoneutrality, housed at RT, and in mice exposed to 4^0C for 4 hours. 36b4 was used for internal normalization. Statistical difference between groups by unpaired 2-tailed T-test, n.s. non significant, *$p< 0.05$, **$p< 0.01$ and *** $p<0.001$ **(A-F)**.

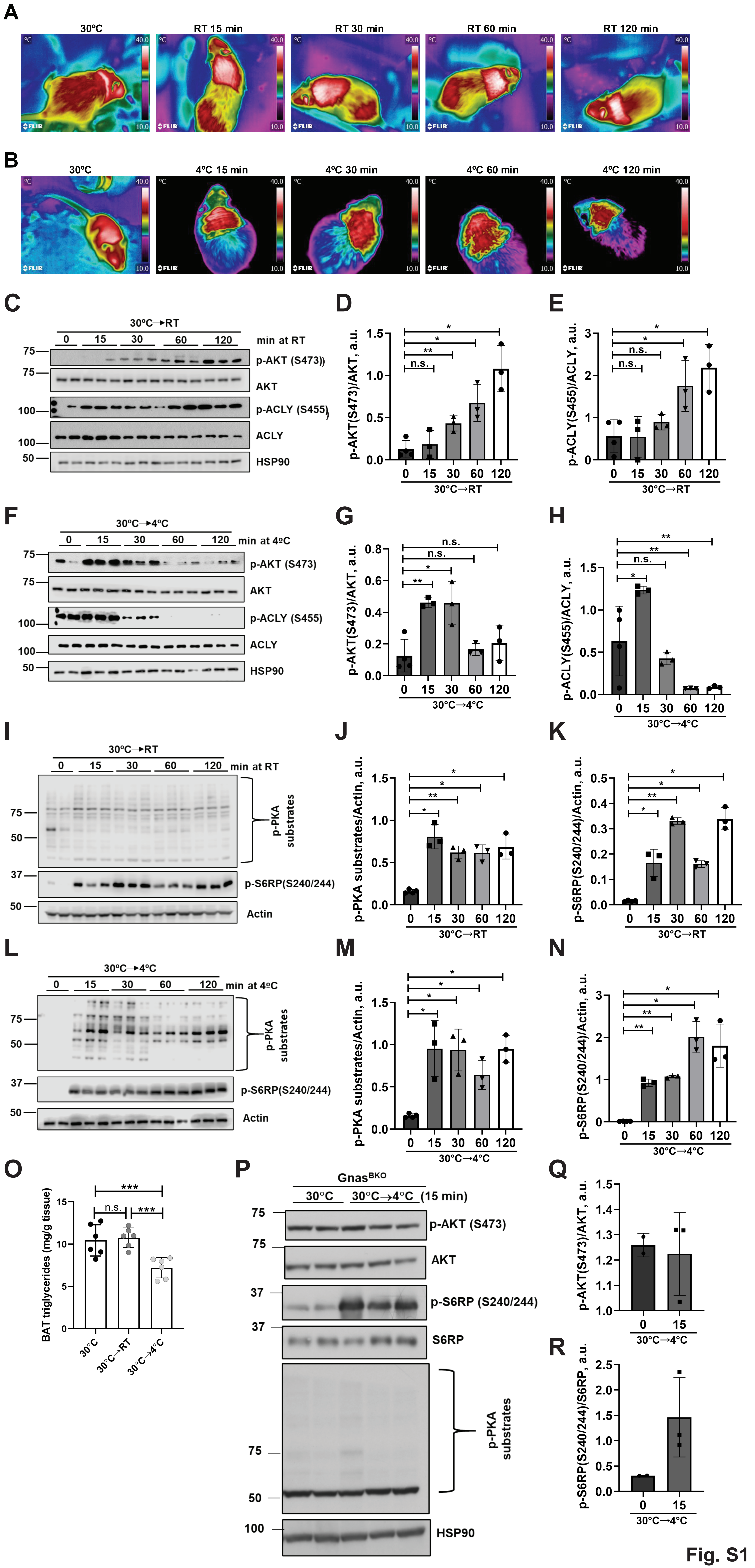
A
30ºC
RT 15 min
RT 30 min
RT 60 min
RT 120 min
B
30ºC
4ºC 15 min
4ºC 30 min
4ºC 60 min
4ºC 120 min
C
30ºC→RT
min at RT
p-AKT (S473)
AKT
p-ACLY (S455)
ACLY
HSP90
D
p-AKT(S473)/AKT, a.u.
30°C→RT
E
p-ACLY(S455)/ACLY, a.u.
30°C→RT
F
30ºC→4ºC
min at 4ºC
p-AKT (S473)
AKT
p-ACLY (S455)
ACLY
HSP90
G
p-AKT(S473)/AKT, a.u.
30°C→4°C
H
p-ACLY(S455)/ACLY, a.u.
30°C→4°C
I
30ºC→RT
min at RT
p-PKA substrates
p-S6RP(S240/244)
Actin
J
p-PKA substrates/Actin, a.u.
30°C→RT
K
p-S6RP(S240/244)/Actin, a.u.
30°C→RT
L
30ºC→4ºC
min at 4ºC
p-PKA substrates
p-S6RP(S240/244)
Actin
M
p-PKA substrates/Actin, a.u.
30°C→4°C
N
p-S6RP(S240/244)/Actin, a.u.
30°C→4°C
O
BAT triglycerides (mg/g tissue)
30°C
30°C→RT
30°C→4°C
P
Gnas^BKO
30°C
30°C→4°C (15 min)
p-AKT (S473)
AKT
p-S6RP (S240/244)
S6RP
p-PKA substrates
HSP90
Q
p-AKT(S473)/AKT, a.u.
30°C→4°C
R
p-S6RP(S240/244)/S6RP, a.u.
30°C→4°C
Fig. S1

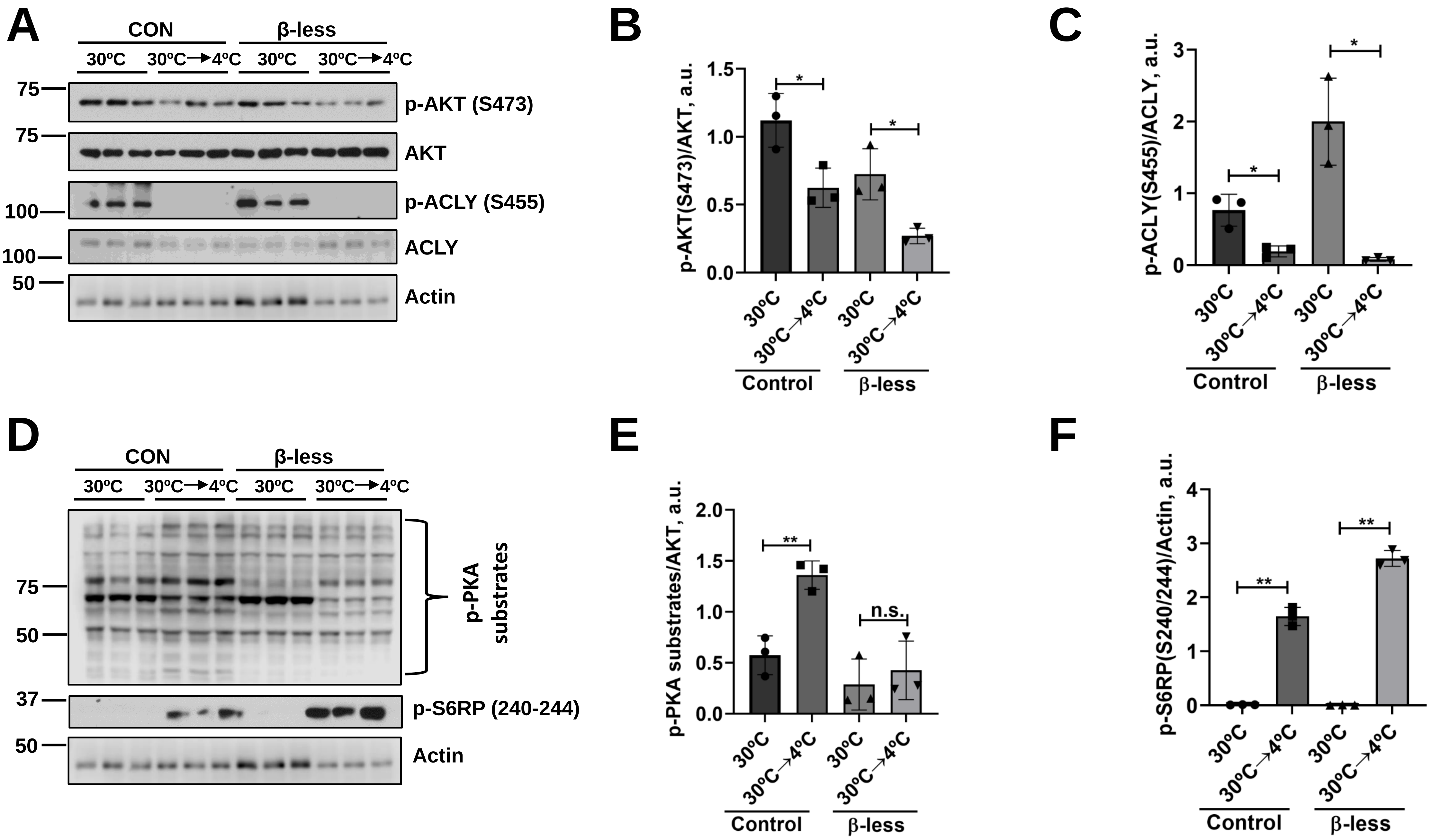
A
CON
β-less
30ºC
30ºC→4ºC
p-AKT (S473)
AKT
p-ACLY (S455)
ACLY
Actin
75
100
50
B
p-AKT(S473)/AKT, a.u.
0.0
0.5
1.0
1.5
*
Control
β-less
C
p-ACLY(S455)/ACLY, a.u.
0
1
2
3
D
p-PKA substrates
p-S6RP (240-244)
37
E
p-PKA substrates/AKT, a.u.
2.0
**
n.s.
F
p-S6RP(S240/244)/Actin, a.u.
4

Fig. S2

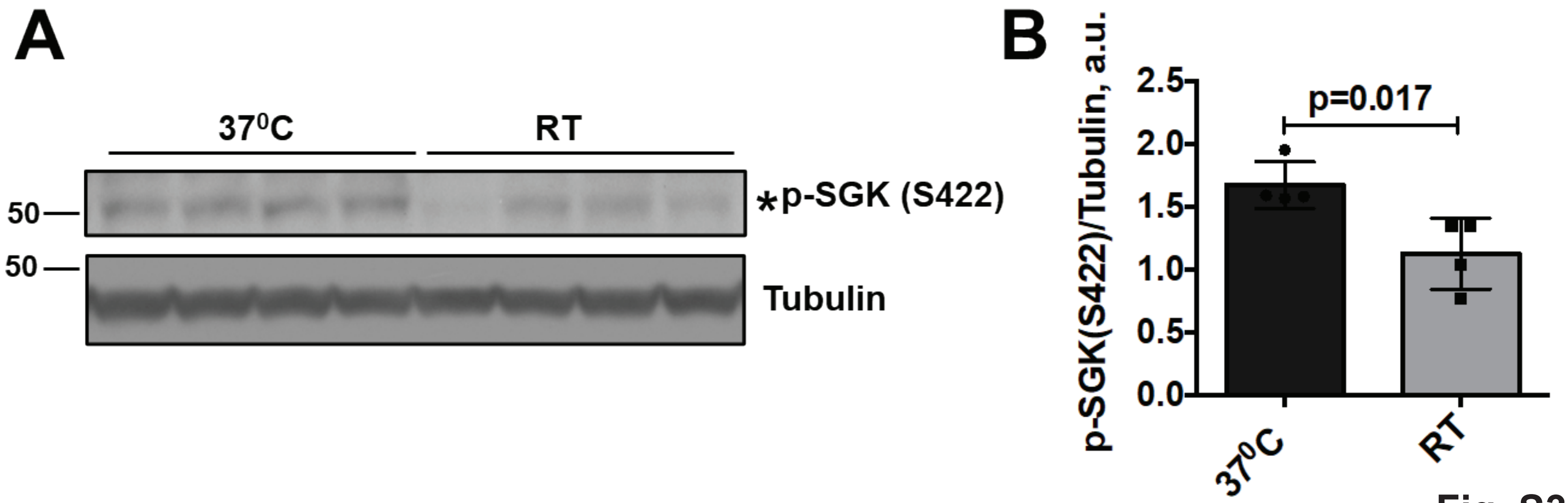
A
37⁰C
RT
50
50
*p-SGK (S422)
Tubulin
B
p-SGK(S422)/Tubulin, a.u.
2.5
2.0
1.5
1.0
0.5
0.0
p=0.017
37⁰C
RT

Fig. S3

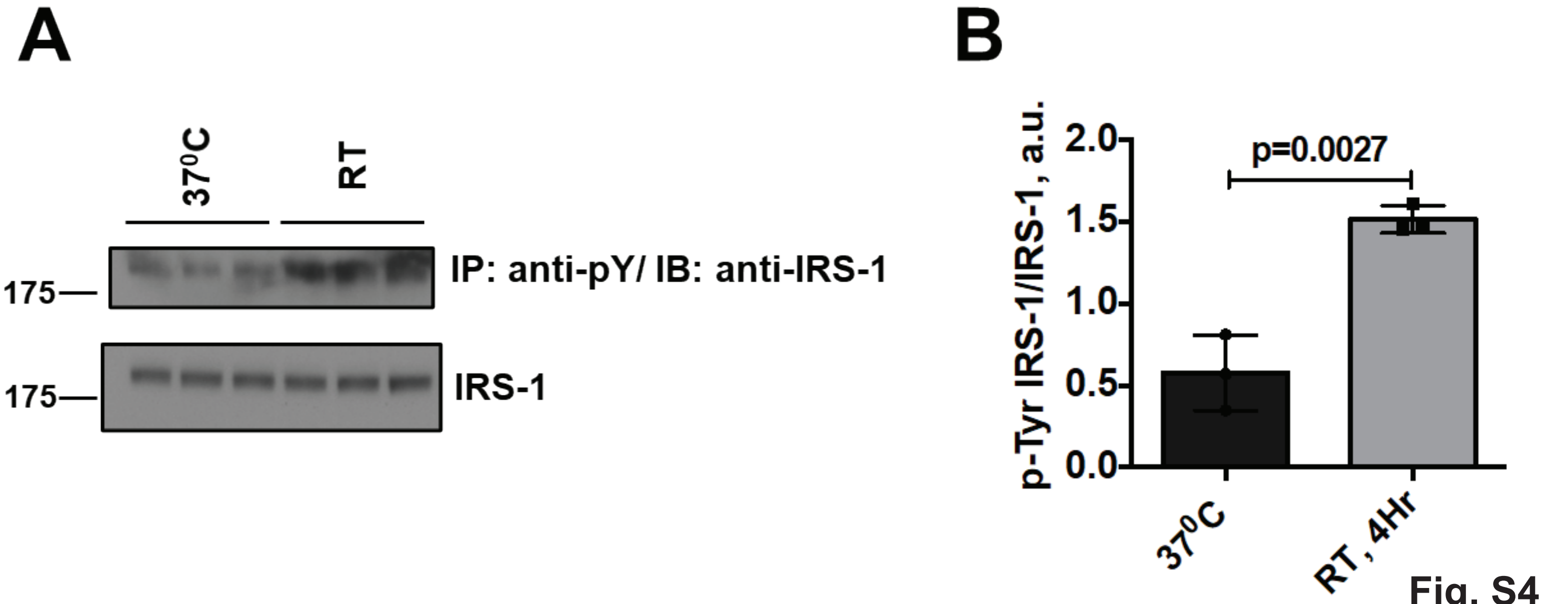
A
37⁰C
RT
175
IP: anti-pY/ IB: anti-IRS-1
175
IRS-1
B
p-Tyr IRS-1/IRS-1, a.u.
2.0
1.5
1.0
0.5
0.0
p=0.0027
37⁰C
RT, 4Hr

Fig. S4

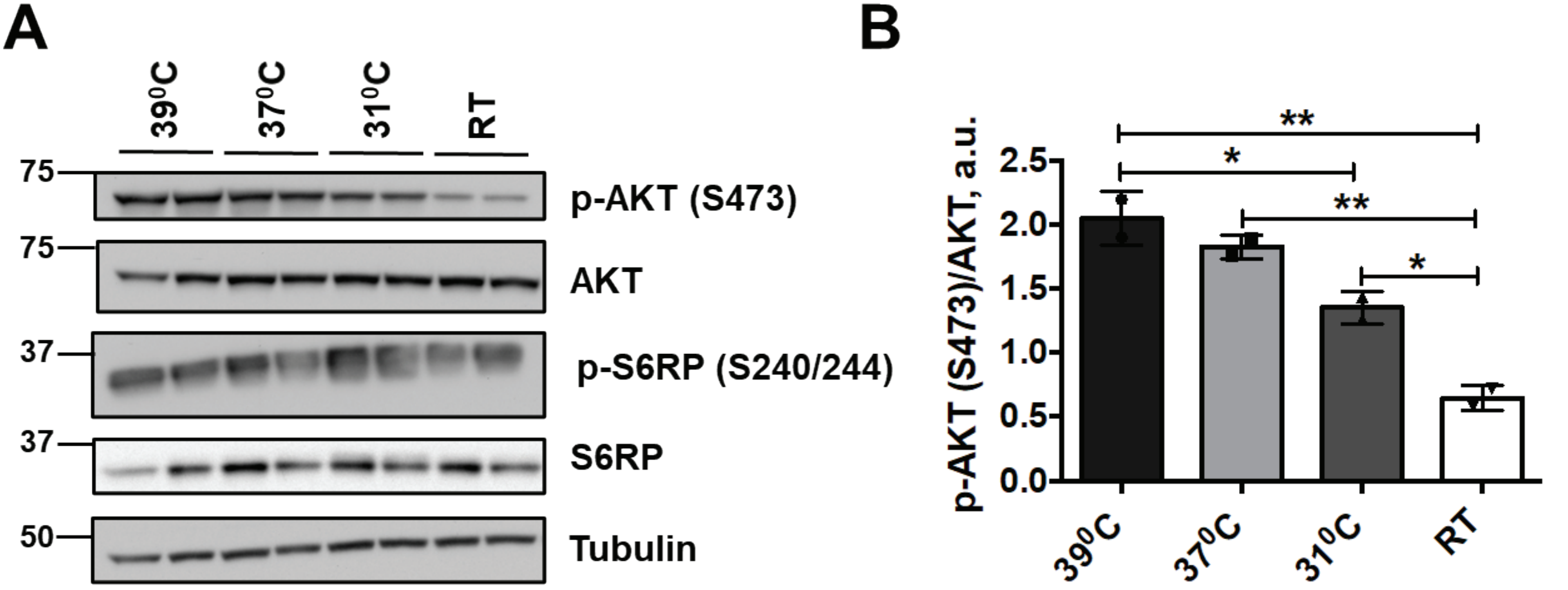
A
39⁰C
37⁰C
31⁰C
RT
75
75
37
37
50
p-AKT (S473)
AKT
p-S6RP (S240/244)
S6RP
Tubulin
B
p-AKT (S473)/AKT, a.u.
2.5
2.0
1.5
1.0
0.5
0.0
**
*
**
*
39⁰C
37⁰C
31⁰C
RT

Fig. S5

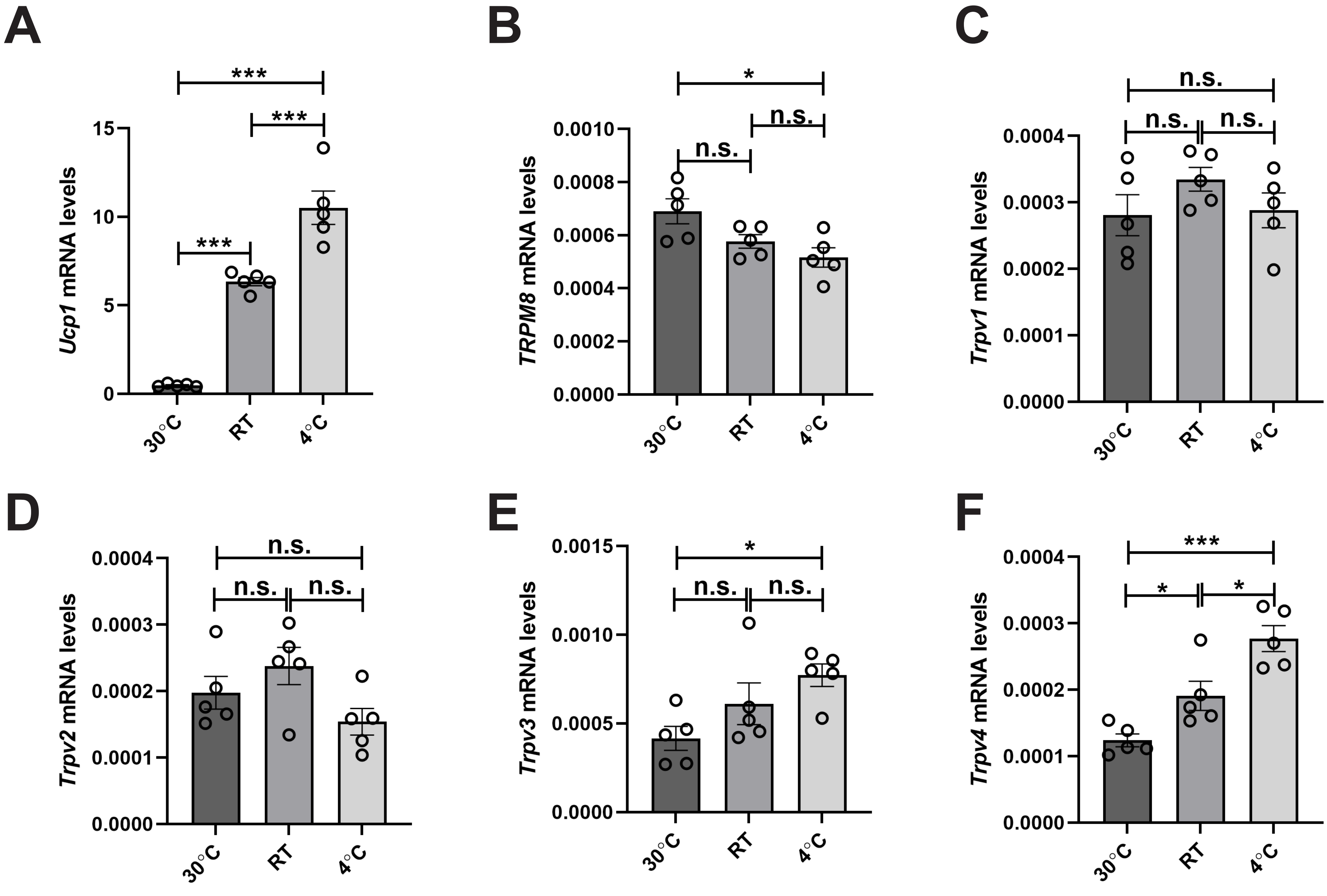
A
B
C
D
E
F
Ucp1 mRNA levels
TRPM8 mRNA levels
Trpv1 mRNA levels
Trpv2 mRNA levels
Trpv3 mRNA levels
Trpv4 mRNA levels
30°C
RT
4°C
***
*
n.s.
0
5
10
15
0.0000
0.0002
0.0004
0.0006
0.0008
0.0010
0.0001
0.0003
0.0005
0.0015

Fig. S6
